# Synergistic infection of two viruses MCMV and SCMV increases the accumulations of both MCMV and MCMV-derived siRNAs in maize

**DOI:** 10.1038/srep20520

**Published:** 2016-02-11

**Authors:** Zihao Xia, Zhenxing Zhao, Ling Chen, Mingjun Li, Tao Zhou, Congliang Deng, Qi Zhou, Zaifeng Fan

**Affiliations:** 1 State Key Laboratory of Agro-biotechnology and Ministry of Agriculture Key Laboratory for Plant Pathology, China Agricultural University, Beijing 100193, China; 2 Beijing Entry-exit Inspection and Quarantine Bureau, Beijing 100026, China; 3 Chinese Society of Inspection and Quarantine, Beijing 100026, China

## Abstract

The co-infection of *Maize chlorotic mottle virus* (MCMV) and *Sugarcane mosaic virus* (SCMV) can cause maize lethal necrosis. However, the mechanism underlying the synergistic interaction between these two viruses remains elusive. In this study, we found that the co-infection of MCMV and SCMV increased the accumulation of MCMV. Moreover, the profiles of virus-derived siRNAs (vsiRNAs) from MCMV and SCMV in single- and co-infected maize plants were obtained by high-throughput sequencing. Our data showed that synergistic infection of MCMV and SCMV increased remarkably the accumulation of vsiRNAs from MCMV, which were mainly 22 and 21 nucleotides in length. The single-nucleotide resolution maps of vsiRNAs revealed that vsiRNAs were almost continuously but heterogeneously distributed throughout MCMV and SCMV genomic RNAs, respectively. Moreover, we predicted and annotated dozens of host transcript genes targeted by vsiRNAs. Our results also showed that maize *DCLs* and several *AGOs* RNAs were differentially accumulated in maize plants with different treatments (mock, single or double inoculations), which were associated with the accumulation of vsiRNAs. Our findings suggested possible roles of vsiRNAs in the synergistic interaction of MCMV and SCMV in maize plants.

RNA silencing is a conserved mechanism in most eukaryotic organisms that regulates the expression of endogenous genes and counteracts invading nucleic acids, including viruses^1^,^2^,^3^. As a defence against viruses in plants, RNA silencing is triggered by double-stranded RNA (dsRNA) from replication intermediates as well as highly structured single-stranded RNA (ssRNA), which can be recognized and cleaved into virus-derived small interfering RNAs (vsiRNAs) of 21–24 nucleotides (nt) by DICER-like (DCL) proteins^4^,^5^. These vsiRNAs are then incorporated into RNA-induced silencing complexes (RISCs) containing Argonaute (AGO) proteins, which are the core components of the complexes, targeting the viral RNAs and host mRNAs in a sequence-specific manner mainly by cleavage^6^,^7^,^8^,^9^. In plants, cellular RNA-dependent RNA polymerases (RDRs) can amplify the effect of RNA silencing by converting aberrant RNAs to dsRNAs and producing secondary vsiRNAs^10^,^11^. To counteract RNA silencing defence mechanisms, plant viruses express viral suppressors of RNA silencing (VSRs), which may affect the RNA silencing machinery at multiple steps^1^,^12^.

Plants encode multiple DCL, AGO and RDR proteins involved in antiviral defences^3^,^13^. DCL4 and DCL2 play essential roles in defence against distinct (+)-strand RNA viruses in a hierarchical and redundant manner^11^,^13^,^14^,^15^,^16^. In virus infected plants, DCL4 is the major component in vsiRNAs production and produces the most abundant 21-nt vsiRNAs; in the absence of DCL4, DCL2 generates 22-nt vsiRNAs as a surrogate^11^,^15^,^16^,^17^. However, DCL2-dependent 22-nt vsiRNAs are less efficient in mediating antiviral silencing and the antiviral activities of DCL4 and DCL2 have tissue specificity^18^,^19^. DCL3 generates 24-nt vsiRNAs and plays a role in antiviral defence against DNA viruses as well as DCL1, while their activity of antiviral defences against RNA viruses remains elusive in plants^5^,^15^,^16^,^20^,^21^. In *Arabidopsis thaliana*, a number of AGO proteins have been proved to be involved in antiviral defence by genetic and biochemical criteria^2^,^13^,^14^,^22^. AGO1, AGO2 and AGO7 contribute to the defence against various viruses while an *ago1* mutant is less susceptible to *Tobacco rattle virus* (TRV), to which *ago4* mutant is more susceptible^9^,^21^,^23^,^24^,^25^. Recent studies have also revealed that AGO5 plays a role in antiviral RNA silencing^26^. One model states that vsiRNAs are recruited into specific AGO complexes to function in antiviral silencing, which is preferentially, but not exclusively, dictated by their 5′-terminal nucleotides^2^,^12^,^22^,^27^,^28^. AGO1, 2, 3, 5, 7 and 10 can bind to vsiRNAs and exhibit *in vitro* slicer activity^29^. Moreover, AGO1, 2, 3, 4, 5 and 9 can all bind to siRNAs derived from viruses or viroids *in vivo*, and AGO10 has been demonstrated to associate with siRNAs derived from VSR-deficient *Turnip mosaic virus* (TuMV)^18^,^28^,^30^,^31^. Interestingly, it has recently been reported that AGO18, a member of a monocot-specific AGO protein clade, confers broad-spectrum virus resistance in rice by sequestering a host microRNA and is induced in virus-infected tissues^32^. One or more of RDR1, RDR2, and RDR6 have been shown to be involved in antiviral silencing by amplification of secondary vsiRNAs and exhibit specificity in targeting viral genome sequences^10^,^11^,^18^,^20^,^21^,^33^.

In addition to targeting viral RNAs, vsiRNAs have been predicted to target host mRNAs at post-transcriptional level using bioinformatics and a few studies have provided the experimental evidence^5^,^6^,^7^,^8^,^33^,^34^,^35^. It has been reported that the *Arabidopsis* At1g76950 mRNA can be down-regulated by vsiRNA derived from the *Cauliflower mosaic virus* 35S leader sequence and At1g30460 and At2g16595 mRNAs were specifically cleaved by vsiRNAs from *Tobacco mosaic virus* (Cg)^5^,^33^. In addition, two research groups simultaneously demonstrated that the Y-satellite of CMV produced a vsiRNA that could specifically and directly silence the *ChlI* gene in *Nicotiana benthamiana* and induce yellow symptoms^7^,^8^. Moreover, the chloroplastic heat-shock protein 90 mRNAs were targeted by the siRNA containing the pathogenic determinant of a chloroplast-replicating^34^. By degradome analysis and 5′ RACE, several host mRNAs were proved to be silenced by vsiRNAs from *Grapevine fleck virus* and *Grapevine rupestris stem pitting-associated virus* in a sequence-specific manner^35^. Recently, the tomato *callose synthase* genes were reported to be silenced by a small RNA derived from the virulence-modulating region of the *Potato spindle tuber viroid*^36^.

*Maize chlorotic mottle virus* (MCMV) in the genus *Machlomovirus* of the family *Tombusviridae* can infect various crops and lead to typical symptoms, such as mild mosaic, severe stunting, and leaf necrosis^37^,^38^,^39^. Maize lethal necrosis (MLN) is caused by the synergistic infection between MCMV and *Maize dwarf mosaic virus* (MDMV), *Wheat streak mosaic virus* (WSMV) or *Sugarcane mosaic virus* (SCMV), leading to serious yield losses in maize (*Zea mays* L.)^40^,^41^,^42^. The effect reported for these synergisms is a dramatic increase in MCMV concentrations in mix-infected plants compared with single-infected plants^40^,^41^. However, the mechanism underlying the synergistic interaction between MCMV and SCMV remains elusive. In this study, we found that the synergistic infection of MCMV and SCMV increased the accumulation of MCMV. Moreover, we obtained the profiles of vsiRNAs from SCMV and MCMV in singly and doubly infected maize plants by high-throughput sequencing, respectively. The characters of vsiRNAs were analysed and the target genes of some vsiRNAs were predicted and annotated. In addition, we investigated the gene expression of maize *DCLs* and several *AGOs* through the characterization of the mRNA accumulation in phosphate buffer (mock), SCMV, MCMV or co-inoculated (S + M) maize plants.

## Results

### Synergistic infection of MCMV and SCMV increases the accumulation of MCMV

**Figure 1:**
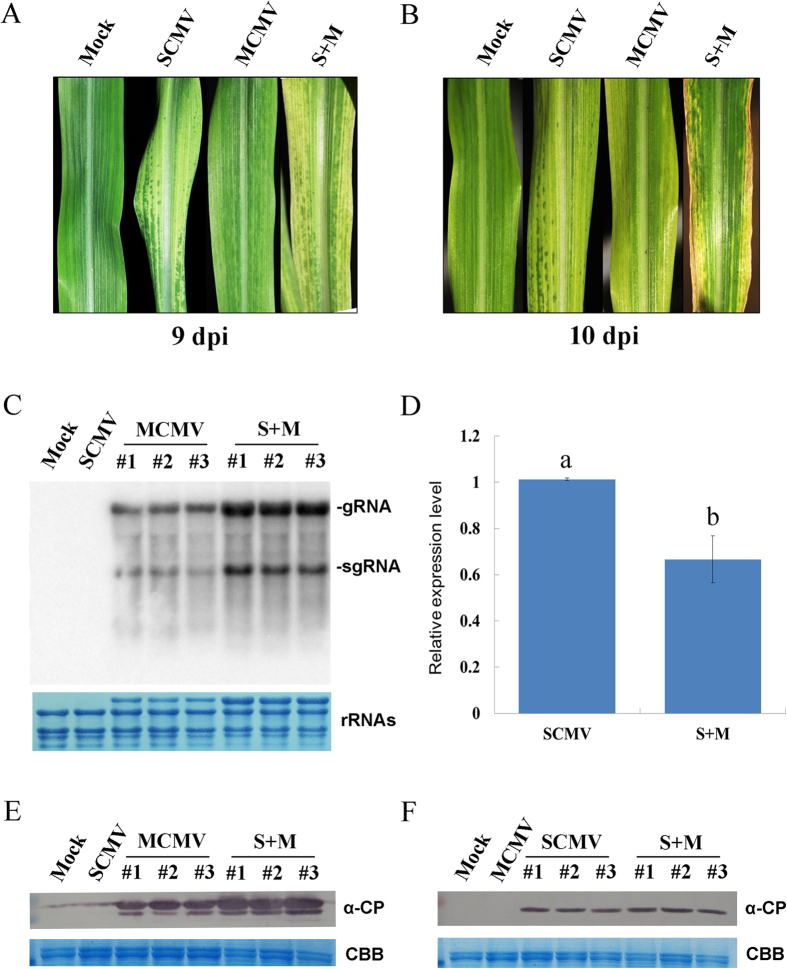
Co-infection of SCMV and MCMV increased the accumulation of MCMV. (**A**,**B**) The symptoms of the first systemically infected leaves at 9 and 10 dpi, respectively. (**C**) The accumulations of MCMV genome were determined by Northern blotting at 9 dpi in buffer (Mock), SCMV, MCMV and S + M inoculated maize plants. Three independent MCMV and S + M infected maize plants were used, and Mock and SCMV inoculated plants were used as controls. Methylene blue staining (bottom panel) of the same extracts was shown to demonstrate equal loading. (**D)** The relative expressions of SCMV RNAs were determined by qRT-PCR at 9 dpi in SCMV and S + M infected maize plants. Three independent experiments were conducted with at least 3 biological replicates each and the data were analysed using a two-sample *t*-test. Bars represented the grand means ± SD. Different letters in lowercase indicate a significant difference (*P*-value < 0.05). (**E**,**F**) The accumulation levels of MCMV and SCMV CP, respectively. Western blotting was performed using the systemically infected leaves of buffer (Mock), SCMV, MCMV or S + M inoculated maize plants at 9 dpi. Coomassie brilliant blue (CBB) staining (bottom panel) of the same extracts was shown to demonstrate equal loading.

To understand the synergistic interaction of MCMV and SCMV, maize seedlings at the third leaf stage were inoculated with phosphate buffer (Mock), SCMV, MCMV and both viruses (S + M), respectively. The first systemically infected leaves of co-infection became significantly chlorotic at 9 days post inoculation (dpi) and developed necrotic areas at 10 dpi while the leaves of SCMV or MCMV single infection showed mosaic or chlorotic symptom at 9 or 10 dpi, respectively (Fig. 1A,B). Total RNA was isolated from the systemically infected leaves for Northern blotting and quantitative reverse transcription (RT)-PCR (qRT-PCR) analyses at 9 dpi. The accumulation level of MCMV genomic RNAs was higher in mix-infected leaves than that in single-infected leaves (Fig. 1C), while the SCMV RNA was slightly decreased (Fig. 1D). The expression levels of SCMV and MCMV CP were quantified by Western blotting. The results showed that MCMV CP accumulated to higher levels in mix-infected leaves as did the genomic RNAs (Fig. 1E). However, there was no obvious difference in the expression level of SCMV CP between mix-infected and single-infected leaves (Fig. 1F). Taken together, synergistic infection increased the expression levels of both the genomic RNAs and coat protein of MCMV, but decreased the accumulation of SCMV RNA.

### Profiles of vsiRNAs in SCMV-, MCMV- or co-inoculated maize plants

**Figure 2:**
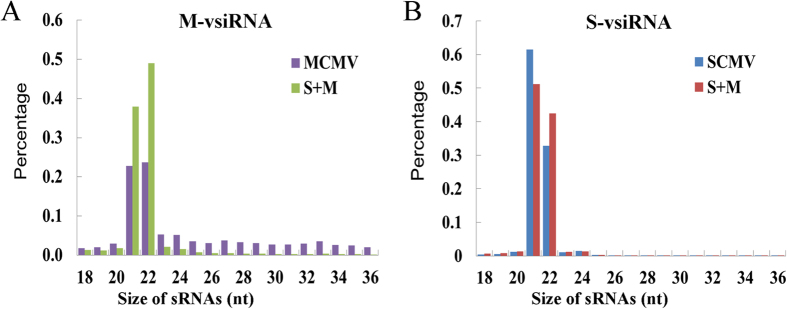
Size distribution of vsiRNAs. (**A**) Size distribution of M-vsiRNAs in MCMV and S + M inoculated maize plants. (**B**) Size distribution of S-vsiRNAs in SCMV and S + M inoculated maize plants.

To obtain the profiles of vsiRNAs produced during viral infections, total RNA was extracted from the systemically infected leaves of maize plants inoculated with buffer (Mock), SCMV, MCMV and S + M at 9 dpi, respectively. The cDNA libraries of small RNAs generated from the total RNA were analysed by high-throughput sequencing on the Illumina Solexa platform yielding around 10,000,000 sequences for each library (Table 1). Reads ranging from 18- to 36-nt were mapped to the viral genomes within 2 mismatches in both sense and antisense orientations. A total of 1,255,641 and 6,740,592 vsiRNA reads were obtained in MCMV- and SCMV-inoculated maize plants, respectively, and 2,044,540 MCMV-derived siRNA (M-vsiRNA) and 6,520,905 SCMV-derived siRNA (S-vsiRNA) reads were obtained in S + M inoculated maize plants (Table 1). Further analysis showed that 22- and 21-nt M-vsiRNAs accumulated to higher levels in S + M inoculated maize plants than that in MCMV inoculated maize plants, which accounted for 87.06% and 46.51% of total M-vsiRNAs, respectively (Fig. 2A). Moreover, the percentage of 22-nt M-vsiRNAs was higher than that of 21-nt M-vsiRNAs in both MCMV and S + M inoculated maize plants (23.73% compared with 22.79%, and 49.07% compared with 37.98%, respectively) (Fig. 2A). The majority of S-vsiRNAs were 21 nt and 22 nt in length in both SCMV and S + M inoculated maize plants, representing 94.19% and 93.54% of total S-vsiRNAs, respectively, and the 21-nt S-vsiRNAs accumulated more than 22-nt S-vsiRNAs (Fig. 2B). Although the total percentage of 21-nt and 22-nt S-vsiRNAs was almost equal, the accumulation of 21-nt S-vsiRNAs decreased while 22-nt S-vsiRNAs increased in S + M inoculated maize plants compared with that in SCMV inoculated maize plants (51.13% compared with 61.44%, and 42.42% compared with 32.75%, respectively) (Fig. 2B). These results showed that synergistic infection increased the accumulation of M-vsiRNAs of both 21 nt and 22 nt and impacted the proportion of S-vsiRNAs of both 21 nt and 22 nt.

### Analysis of the strand polarity and 5′-terminal nucleotide of vsiRNAs

**Figure 3:**
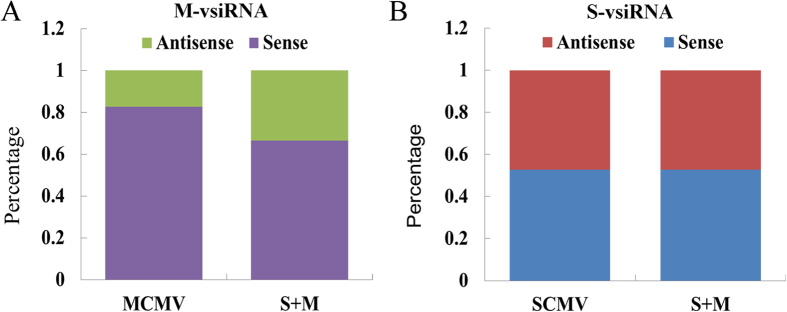
Percentage distribution of vsiRNAs with respect to strand polarity. **(A**) The strand polarity of M-vsiRNAs in MCMV and S + M inoculated maize plants. (**B**) The strand polarity of S-vsiRNAs in SCMV and S + M inoculated maize plants.

To understand the origin of the vsiRNAs, we analysed the strand polarity of vsiRNAs. For M-vsiRNAs, a clear prevalence for (+)-sense strand was observed in MCMV and S + M inoculated maize plants, accounting for 82.80% and 66.53% of total M-vsiRNAs, respectively (Fig. 3A). However, there were no obvious differences in strand polarity for S-vsiRNAs in SCMV and S + M inoculated maize plants (Fig. 3B). Moreover, co-infection of SCMV and MCMV increased the accumulation of M-vsiRNAs from (−)-sense strand of MCMV, but had no obvious effect on S-vsiRNAs (Fig. 3A,B)

**Figure 4:**
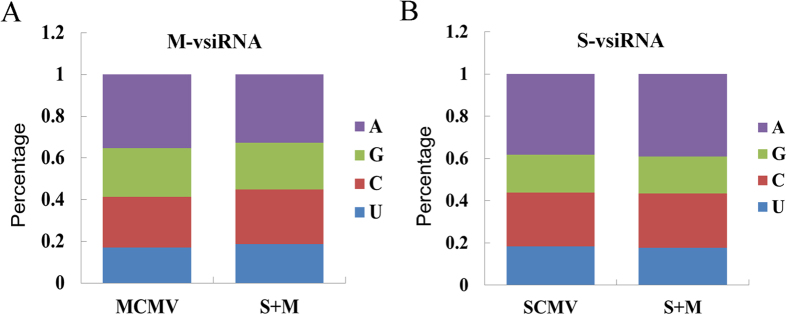
5′-terminal nucleotide abundance of vsiRNAs. **(A**) 5′-terminal nucleotide abundance of M-vsiRNAs in MCMV and S + M inoculated maize plants. (**B**) 5′-terminal nucleotide abundance of S-vsiRNAs in SCMV and S + M inoculated maize plants.

Previous studies have demonstrated that the 5′-terminal nucleotides of small RNAs mainly determined the recruitment by specific AGOs^27^,^28^. To explore the potential interactions between vsiRNAs and distinct AGO complexes, the nucleotide at the 5′-terminal position in vsiRNA sequences was analysed. There was an A preference existing in the 5′-terminal nucleotides of M-vsiRNAs in MCMV and S + M inoculated maize plants, which were mainly recruited by AGO2 and/or AGO4, although the percentage was slightly decreased in the co-infected maize plants (32.61% compared with 35.28%)(Fig. 4A). Like in M-vsiRNAs, A was the most abundant at the 5′-terminal nucleotide of S-vsiRNAs and there was no obvious difference in SCMV and S + M inoculated maize plants (Fig. 4B). Interestingly, vsiRNAs with 5′-terminal U, which were mainly loaded into AGO1, accounted for a low percentage (17–19%) of total vsiRNAs (Fig. 4).

### Mapping vsiRNAs along SCMV and MCMV genomic RNAs

**Figure 5:**
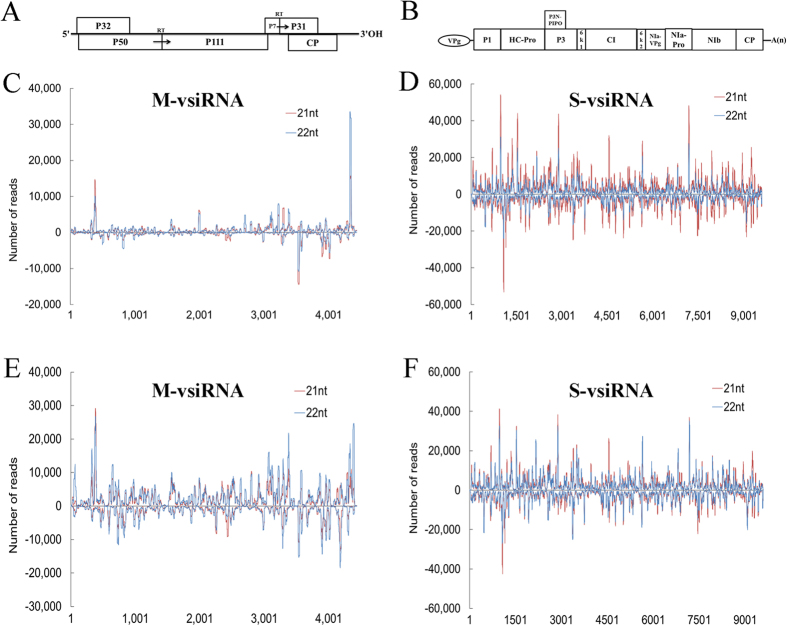
The single-nucleotide resolution maps of 21- and 22-nt vsiRNAs. (**A**) Schematic diagram of the MCMV genome. (**B**) Schematic diagram of the SCMV genome. The single-nucleotide resolution maps of 21- and 22-nt M-vsiRNAs along the MCMV genome in MCMV (**C**) and S + M (**E**) inoculated maize plants and 21- and 22-nt S-vsiRNAs along the SCMV genome in SCMV (**D**) and S + M (**F**) inoculated maize plants. The bars above the axis represent the reads of vsiRNAs from the viral genomic strand starting at the respective positions; the bars below represent the reads of vsiRNAs from the complementary strand of viral genomes ending at the respective positions.

To gain further insight into the origin of the vsiRNAs, 21- and 22-nt vsiRNA sequences were mapped along the (+)- and (−)-sense strands of SCMV and MCMV genomes, respectively. The single-nucleotide resolution maps indicated that vsiRNAs were almost continuously but heterogeneously distributed throughout the (+)- and (−)-sense strands of SCMV and MCMV genomes, respectively (Fig. 5). In MCMV-infected maize plants, one obvious hotspot corresponding to the 3′-UTR region was distributed in the (+)-sense strand of MCMV genome (Fig. 5A,C). As co-infection increased the accumulation of 21- and 22-nt M-vsiRNAs, the hotspots distributed in the (+)- and (−)-sense strands of MCMV genome were both increased in co-infected maize plants, which mainly located in the P32/P50, P31 and CP coding regions and the 3′-UTR region (Fig. 5E). There were no obvious differences in the distribution of S-vsiRNAs along the (+)- and (−)-sense strands of SCMV genome between SCMV and S + M inoculated maize plants, except that the 22-nt S-vsiRNAs were presented more in S + M inoculated maize plants (Fig. 5D,F). Further estimation of the hotspots generated by S-vsiRNAs showed that the HC-Pro coding region had a tendency to produce higher levels of S-vsiRNAs in SCMV and S + M inoculated maize plants (Fig. 5B,D,F).

### Prediction and annotation of the putative target genes of vsiRNAs

**Figure 6:**
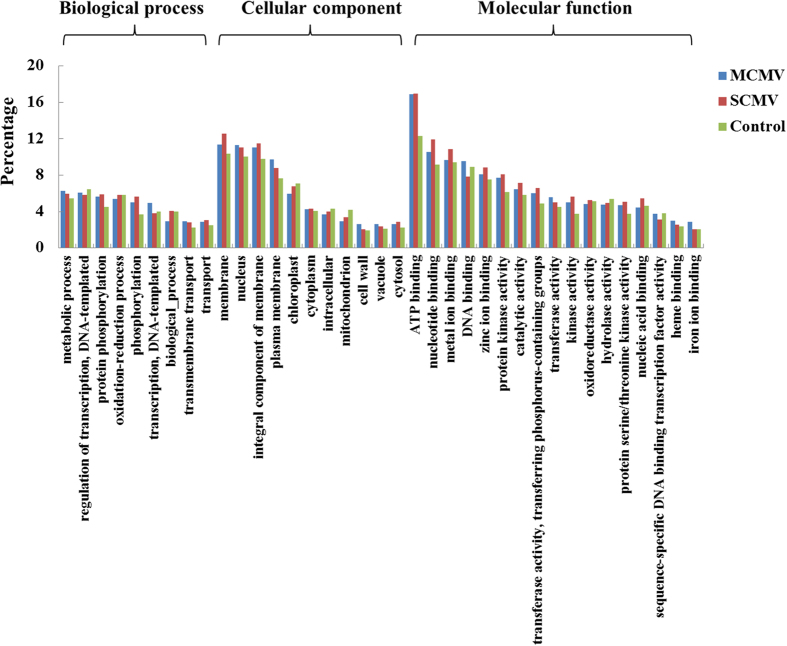
GO classification of the predicted target genes of vsiRNA derived from MCMV and SCMV in maize. The vsiRNA target genes were assigned using Blast2GO. “Control” indicates the percentage of the genes with specific category in all GO-annotated maize genes.

The putative target genes of vsiRNAs were predicted using the MiRnada, which was an algorithm for finding the targets of miRNAs^43^. In our study, only these vsiRNAs with high abundant reads (top 50 derived from either genomic strand or complementary strand) were selected to predict target genes in co-infected maize plants (Supplementary Table S1). Thousands of target genes were predicted and only those whose scores were not less than 180 were presented (Supplementary Table S2). Moreover, we selected the putative target genes whose scores were not less than 170 for further analyses. To understand the putative roles of the predicted target genes of vsiRNAs in maize, we conducted Gene Ontology (GO) analysis using Blast2GO in terms of biological processes, cellular components, and molecular functions^44^. A total of 1969 and 1560 predicted target genes of M-vsiRNAs and S-vsiRNAs were annotated by GO analysis, respectively. The percentage of the predicted target genes with a particular category in the total GO-annotated target genes was indicated in Fig. 6. Interestingly, the major categorized groups of the predicted target genes of vsiRNAs were similar for both MCMV and SCMV. ‘Metabolic process’ was the most highly represented group under the biological process category, followed by ‘regulation of transcription, DNA-templated’, ‘protein phosphorylation’ and ‘oxidation-reduction process’. For the cellular component category, ‘membrane’, ‘nucleus’ and ‘integral component of membrane’ were the most highly represented groups. With regard to the molecular function category, ‘binding’ groups were the most highly represented, mainly including ‘ATP binding’, ‘nucleotide binding’, ‘metal ion binding’ and ‘DNA binding’. To demonstrate whether or not the predicted target genes with a particular category were enriched, we used the percentage of the genes with specific category in all GO-annotated maize genes as the control.

**Figure 7:**
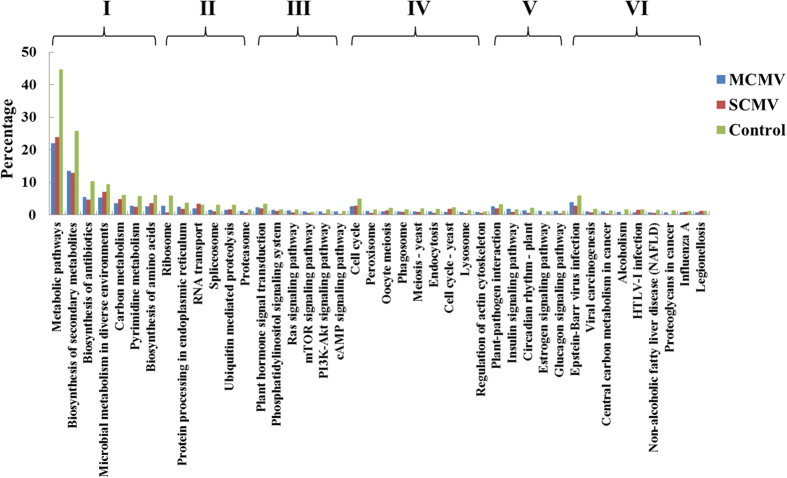
KEGG classification of the predicted target genes of vsiRNAs derived from MCMV and SCMV in maize. The vsiRNA target genes were assigned based on the KEGG database using BLASTx. I: Metabolism; II: Genetic Information Processing; III: Environmental Information Processing; IV: Cellular Processes; V: Organismal Systems; VI: Human Diseases. “Control” means the percentage of the genes with specific category in all KEGG-annotated maize genes.

To get a better understanding of the special biochemical pathways for the predicted target genes of vsiRNAs, we assigned them based on the KEGG database using BLASTx^45^. A total of 816 and 674 predicted target genes of M-vsiRNAs and S-vsiRNAs were aligned in the KEGG database, respectively, and the percentage of the predicted target genes with a specific category were shown (Fig. 7). The metabolism pathway contained most of the predicted target genes of vsiRNAs for MCMV and SCMV, in which the most frequently represented pathway was ‘Metabolic pathways’, followed by ‘Biosynthesis of secondary metabolites’. To understand the enrichment of the predicted target genes in a particular category, the percentage of the genes with specific category in all KEGG-annotated maize genes was used as the control.

### Northern blotting confirmed that the co-infection of SCMV and MCMV increased the accumulation of M-vsiRNAs

**Figure 8:**
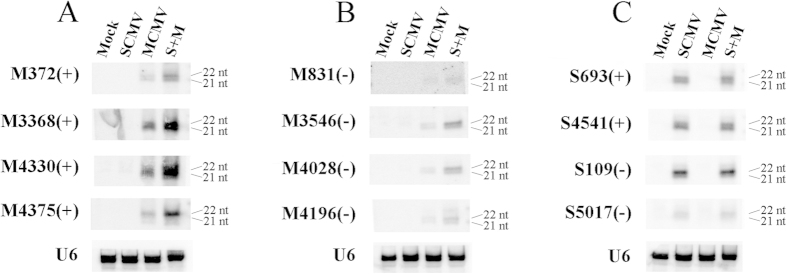
Northern blotting analysis of vsiRNAs. **(A**,**B**) Northern blotting analysis of M-vsiRNAs in MCMV and S + M inoculated maize plants. (**C**) Northern blotting analysis of S-vsiRNAs in SCMV and S + M inoculated maize plants. The “M” means the vsiRNAs from MCMV and “S” means that from SCMV. The numbers represent vsiRNAs starting positions of the (+)-sense strand or ending positions of the (−)-sense strand of viral genomes. “(+)” indicates vsiRNAs derived from (+)-sense strand of viral genomes and “(−)” indicates the (−)-sense strand. “21” and “22” means the positions of 21-nt and 22-nt vsiRNAs, respectively. U6 was used as a loading control.

To confirm the impact of synergistic infection on the production of vsiRNAs, total RNA was extracted to analyse the accumulation of vsiRNAs from different genome positions of both (+)- and (−)-sense strands of SCMV and MCMV by Northern blotting, respectively. The results showed that the vsiRNAs could be detected, although the signal of M831 (−) was weak, which demonstrated the existence of vsiRNAs (Fig. 8). The M-vsiRNAs from S + M co-infected maize plants accumulated more than those from MCMV infected maize plants for M-vsiRNAs derived from both MCMV genomic and complementary strands (Fig. 8A,B), although the results of high-throughput sequencing revealed that the level of M4330 (+) was decreased (Fig. 5C,E). Moreover, for all selected M-vsiRNAs from S + M co-infected maize plants, there was a preference for 22-nt while almost equivalent 21- and 22-nt M-vsiRNAs accumulated in MCMV infected maize plants except for M4375 (+), which had a 22-nt preference (Fig. 8A,B). However, there were no obvious differences in the accumulation of S-vsiRNAs selected between SCMV and S + M infected maize plants (Fig. 8C). For S693 (+) and S4541 (+) from the genomic strand of SCMV, 21- and 22-nt vsiRNAs accumulated almost equally as well as S5017 (−) while S109 (−) had a preference for 22 nt, of which both derived from the complementary strand (Fig. 8C). The results obtained by Northern blotting revealed that synergistic infection increased the accumulation of M-vsiRNAs while had no obvious effect on S-vsiRNAs, which were consistent with that of high-throughput sequencing.

### Differential expression of maize *DCLs* and several *AGOs* mRNAs after SCMV, MCMV or S + M infection

**Figure 9:**
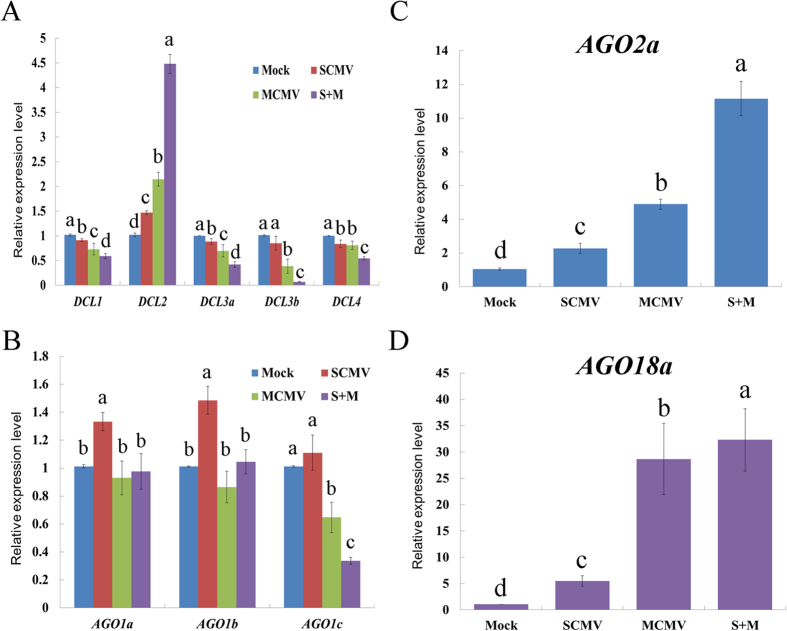
The expression levels of maize *DCLs* and several *AGOs* mRNAs in buffer (Mock), SCMV, MCMV and S + M inoculated maize plants. The expression levels were determined by qRT-PCR at 9 dpi. Three independent experiments were conducted with at least 3 biological replicates each and the data were analysed using a two-sample *t*-test. Bars represented the grand means ± SD. Lowercase letters indicate significant difference (*P*-value < 0.05).

DCLs and AGOs are the most important components of antiviral RNA silencing involved in the biogenesis of vsiRNAs. To investigate the effects of viral infections on the components of RNA silencing, the expression levels of maize *DCLs* and several *AGOs* mRNAs were characterized using qRT-PCR. The results showed that the expression of *DCL2* mRNA was significantly increased in the singly and doubly infected maize plants (Fig. 9A). However, other *DCLs* mRNAs were down-regulated in SCMV, MCMV and S + M infected maize plants except for *DCL3b* in SCMV infection (Fig. 9A). Interestingly, S + M infection had the significant influence on the expression of maize *DCLs* mRNAs: the highest expression level of *DCL2* was detected and other DCLs accumulated to the lowest levels (Fig. 9A). In maize *AGO1* homologs, the expression of *AGO1a* and *AGO1b* were up-regulated while that of *AGO1c* was unchanged in SCMV infected maize plants (Fig. 9B). However, the MCMV and S + M infection decreased the expression of *AGO1c* and had no obvious effect on *AGO1a* and *AGO1b* (Fig. 9B). Moreover, *AGO2a* and *AGO18a* were significantly up-regulated by viral infections, especially in S + M infected maize plants, which accumulated to the highest levels (Fig. 9C,D). These data suggested that viral infections differentially modified the expression of components involved in antiviral RNA silencing pathway.

## Discussion

In plants, synergistic interactions between independent viruses in mixed infections have been well documented^46^, but the mechanism underlying these interactions remains elusive. In our study, the expression levels of MCMV genomic RNAs and CP were increased in S + M co-infected maize plants compared with that in MCMV infected maize plants (Fig. 1C,E), in agreement with the results of previous reports^40^,^41^. It has been demonstrated that HC-Pro, the silencing suppressor encoded by potyviruses, could enhance the pathogenicity and accumulation of other heterologous viruses^46^,^47^,^48^. Moreover, the synergistic infection of WSMV and MCMV was independent of WSMV HC-Pro, which was not a silencing suppressor^48^,^49^. However, the effects of SCMV HC-Pro as well as WSMV P1 on the synergistic infections remain to be investigated, which have been proved to function as suppressors of RNA silencing^50^,^51^.

RNA silencing is a conserved surveillance mechanism in the defence against viruses in plants, which can trigger the production of vsiRNAs in virus-infected plant cells. In this study, the profiles of vsiRNAs from SCMV and MCMV in singly and doubly infected maize plants were obtained to understand the role of RNA silencing in the synergistic interaction between SCMV and MCMV in maize plants. In the SCMV singly or doubly (with MCMV) infected maize plants, S-vsiRNAs accounted for more than half of total small RNAs (Table 1), similar to the results of our previous report^52^. However, the accumulation level of M-vsiRNAs was lower compared with endogenous small RNAs within a library, accounting for 14.75–19.49% of total small RNAs (Table 1). Further analysis of S + M library suggested that there was a preference to SCMV RNAs for RNA silencing, which accumulated more S-vsiRNAs than M-vsiRNAs (Table 1). The nonsense-mediated decay (NMD) was reported to recognize and eliminate viral RNAs with internal termination codons and long 3′-UTRs, but it had no effect on the potyvirus^53^. NMD, as a general virus restriction mechanism in plants, might compete for MCMV RNA substrates with RDR and decrease the accumulation of M-vsiRNAs in MCMV singly and doubly (with SCMV) infected maize plants. In effect, saturation of NMD by increasing amounts of viral RNAs may constitute a switch for RDR action and secondary RNA silencing during viral infection^53^. In co-infected maize plants, the increased accumulation levels of M-vsiRNAs might be the results of processing the increased accumulation of MCMV RNAs by RNA silencing.

In positive-strand RNA virus-infected plants, DCL4-dependent 21-nt vsiRNAs are predominant than DCL2-dependent 22-nt vsiRNAs, which accumulated to higher levels in the absence of DCL4^11^,^15^,^16^,^17^. In the MCMV singly and doubly infected maize plants, 22-nt M-vsiRNAs accumulated to higher levels than 21-nt M-vsiRNAs (Fig. 2A), which might be the results of increased accumulation levels of maize *DCL2* mRNAs and decreased levels of *DCL4* (Fig. 9A). However, the majority of S-vsiRNAs were 21 nt in length in the SCMV singly and doubly infected maize plants (Fig. 2B), indicating that DCL4 played a major role in the biogenesis of S-vsiRNAs and had a preference for processing SCMV RNA, although the accumulation levels of maize *DCL4* mRNAs were down-regulated (Fig. 9A). In the S + M infected maize plants, 22-nt S-vsiRNAs accumulated to higher levels while 21-nt S-vsiRNAs were decreased compared with that in SCMV infected maize plants (Fig. 2B), which associated with the changed accumulation level of maize *DCL4* and *DCL2* mRNAs. The accumulation levels of both 22-nt vsiRNAs and maize *DCL2* mRNAs were increased in the singly and doubly infected maize plants, indicating that DCL2 played an important role in the production of vsiRNAs, which supported the model that cooperative interaction between DCL4 and DCL2 was necessary during systemic antiviral silencing^11^,^19^,^26^. As a result of viral infections, the decreased expression levels of maize *DCL1, DCL3a* and *DCL3b* mRNAs might affect the accumulations of miRNAs and the methylation of DNA and/or histone of maize, however, their roles in the defence against RNA viruses remain to be investigated.

For a long time, the dsRNA replication intermediates were thought to be the major origin of vsiRNAs from positive-strand RNA viruses. However, it has been reported that the vsiRNAs had a positive sense strand bias by high-throughput sequencing, suggesting that vsiRNAs originated predominantly from highly structured single-stranded viral RNAs^4^,^54^. Our results, in agreement with our previous report^52^, demonstrated that almost equal amount of (+)- and (−)-sense S-vsiRNAs existed in SCMV-infected maize plants (Fig. 3B), indicating that most of the S-vsiRNAs were likely generated from dsRNA precursors. In MCMV infected maize plants, the (+)-sense M-vsiRNAs accumulated more than those from the (−)-sense strand (Fig. 3A), suggesting that the majority of M-vsiRNAs were derived from MCMV genomic RNAs. Nevertheless, recent study has revealed that genomic viral RNAs might sequester complementary vsiRNAs during gel electrophoresis^55^, and the sequestration could be decreased by the fully-denaturing formaldehyde polyacrylamide gel electrophoresis (FDF-PAGE)^56^. By applying FDF-PAGE, the predominant precursor of vsiRNAs was demonstrated to be a long dsRNA, however, whether this conclusion is relevant to the origin of M-vsiRNAs remains to be studied.

The small RNAs are loaded into distinct AGO-containing RISCs to function, which are mainly directed by the 5′-terminal nucleotide^27^,^28^. For example, AGO1 preferentially associates with small RNAs that have a 5′ U, AGO2 and AGO4 have a 5′ A preference, and AGO5 mainly associates with small RNAs that begin with a 5′ C. For M-vsiRNAs and S-vsiRNAs, A was the most abundant nucleotide at the 5′-end (Fig. 4), suggesting that these vsiRNAs might be mainly recruited by AGO2 and/or AGO4. Interestingly, the accumulation levels of maize *AGO2* mRNAs were induced in singly and doubly infected maize plants (Fig. 9C), which further increased the possibility that maize AGO2 participated in antiviral defence. Previous reports have shown that AGO1 played a dominant role in the defence against RNA viruses^21^,^23^. However, the accumulation levels of maize *AGO1a* and *AGO1b* mRNAs remained unchanged even *AGO1c* was decreased in MCMV or S + M infected maize plants, although SCMV infection slightly induced the expression of *AGO1a* and *AGO1b* mRNAs (Fig. 9B). Moreover, the vsiRNAs from SCMV and MCMV with a 5′-terminal U accounted for a small proportion (Fig. 4). These data suggested that the AGO1 might play a less important role than AGO2 in the defence against SCMV and MCMV as the results obtained by recent studies^25^,^26^,^31^. In addition, the presence of large amounts of vsiRNAs with 5′-terminal G or C revealed that other AGOs might also be recruited to form specific RISCs and involved in antiviral defence, which were reported to bind siRNAs from viruses or viroids^18^,^28^,^29^,^30^,^31^.

Recent research demonstrated that AGO18, a member of a monocot-specific AGO protein clade, played a role in antiviral defence by sequestering miR168 and was induced in virus-infected tissues^32^. In our study, we detected the expression level of maize *AGO18a* gene, a homolog of rice *AGO18*, by qRT-PCR in buffer (Mock), SCMV, MCMV and S + M inoculated maize plants (the expression level of maize *AGO18b* was almost undetectable in maize leaves^57^). The results showed that the accumulation of *AGO18a* mRNA was significantly induced after viral infections, especially in MCMV and S + M co-infected maize plants (Fig. 9D). We also found that miR168 level was up-regulated in SCMV infected maize plants (Supplementary Fig. S1), in addition to the results that *AGO1a* and *AGO1b* mRNAs were up-regulated (Fig. 9A), suggesting that miR168 could be sequestered by AGO18a as reported previously^32^. Interestingly, the accumulation of miR168 had no obvious change in MCMV or S + M infected maize plants in which the *AGO1a, b, c* mRNAs were not induced (Fig. 9B), suggesting that the significantly induced *AGO18a* might be involved in antiviral defence by other modes of action, such as influencing the function of other miRNAs associated by AGO18^32^. However, the antiviral roles of AGO18 remain to be elucidated in maize plants, especially in MCMV and S + M co-infected maize plants.

## Methods

### Plant growth and virus inoculations

Maize (*Zea mays* L.) inbred line B73 plants were prepared in growth chambers (28 °C day and 22 °C night, 16 h light and 8 h dark cycles) for plant growth and virus inoculation. SCMV-BJ was from previously published sources^58^. MCMV was prepared from the full-length cDNA clone (pMCM41) provided by Dr Kay Scheets. Crude extracts from SCMV or MCMV-infected maize leaf tissues were prepared as described previously^59^, which were then equally mixed as the source of co-infection while equal volume of phosphate buffer was added for single infection, respectively.

### Small RNA sequencing and Bioinformatics analyses

At approximately 9 dpi, before the leaves showed necrosis symptoms as shown in Fig. 1A, the systemically infected leaves were harvested and maintained at −80 °C. With each treatment, the systemically infected leaves of at least 15 maize seedlings were pooled for small RNA sequencing. Total RNA was treated as described^52^ and subjected to Solexa/Illumina sequencing by SBC (Shanghai Biotechnology Corporation, Shanghai, China).

After excluding low quantity reads and 5′- and 3′-adaptor contaminants, the raw reads were obtained. Small RNAs of 18–36 nt in length were extracted and only the sequences identical or complementary to viral genomic sequences within 2 mismatches were recognized as vsiRNAs for further analysis. Small RNA sequences were analysed as described^52^.

### Target Gene Prediction and Analysis

The MiRnada program was used to predict maize mRNAs targeted by vsiRNAs from SCMV and MCMV in co-infected maize plants^43^. The criteria used were as described previously^52^. The predicted target genes were assigned to various GO and KEGG classifications as reported previously^44^,^45^.

### RNA blot analysis

Approximately 40 μg of total RNA was prepared for small RNA blot analysis, and the blots were probed and washed as previously reported^52^. Probe sequences used for small RNA blot analysis were shown in Supplementary Table S3.

About 2 μg of total RNA was used to detect MCMV by Northern blot analyses. Northern blots were performed with [α-^32^P] dCTP randomly-labelled cDNA probes, which were from 3′-terminal 712 nucleotides (3721–4432) of MCMV genome. Random Primer DNA Labelling Kit Ver.2 was used for labelling the probes as instructed by the manufacturer (Takara Bio Inc., Dalian, China). Blots were hybridized at 65^o^ C overnight using hybridisation buffer (Sigma, USA) and washed as instructed by the manufacturer.

### Western blot assay

The protein extraction and Western blot assay were performed as described previously^60^. SCMV CP polyclonal antibody was used at dilution of 1:5000. MCMV CP antibody was kindly provided by Prof. Xueping Zhou (Zhejiang University) and used at dilution of 1:8000.

### Quantitative Real-time RT-PCR

Total RNA was extracted using TRIzol reagent as instructed by the manufacturer (Invitrogen, USA) and treated with RNase-free DNase I (Takara Bio Inc., Dalian, China). About 2 μg of total RNA was used to synthesize the first-strand cDNA with an oligo (dT) primer and the qRT-PCR was performed as previously reported^60^. Maize *UBI* (ubiquitin) was used as an internal standard. The sequence information of maize *DCLs* and *AGOs* was reported in two references^52^,^57^. The information of the primers used in the qRT-PCR experiments were listed in Supplementary Table S4. Three independent experiments were performed with biological and technical replicates.

## Additional Information

**How to cite this article**: Xia, Z. *et al*. Synergistic infection of two viruses MCMV and SCMV increases the accumulations of both MCMV and MCMV-derived siRNAs in maize. *Sci. Rep.* **6**, 20520; doi: 10.1038/srep20520 (2016).

## Supplementary Material

Supplementary Information

## Figures and Tables

**Table 1:** Classification and abundance of small RNAs from buffer (Mock), SCMV, MCMV or S + M inoculated library.

Category	Reads			
	Mock	SCMV	MCMV	S + M
Total raw reads	10,042,093	10,107,781	10,544,484	11,306,497
Clean reads between 18 and 36 nucleotides	8,565,054	9,412,391	8,513,999	10,490,813
MCMV-derived siRNAs within 2 mismatches	–	–	1,255,641	2,044,540
SCMV-derived siRNAs within 2 mismatches	–	6,740,592	–	6,520,905
