# Supplementary material for: Synergistic infection of two viruses MCMV and SCMV increases the accumulations of both MCMV and MCMV-derived siRNAs in maize
Source: Sci Rep. 2016 Feb 11;6:20520. doi: 10.1038/srep20520 (PMC4808907; doi:10.1038/srep20520)
Supplement: Supplementary Information [file srep20520-s1.pdf]

TITLE:

Synergistic infection of two viruses MCMV and SCMV increases the accumulations of both MCMV and MCMV-derived siRNAs in maize

AUTHORS:

Zihao Xia[1], Zhenxing Zhao[1], Ling Chen[1], Mingjun Li[1], Tao Zhou[1], Cong-liang Deng[2], Qi Zhou[3] & Zaifeng Fan[1]*

AFFILIATION:

[1]State Key Laboratory of Agro-biotechnology and Ministry of Agriculture Key Laboratory for Plant Pathology, China Agricultural University, Beijing 100193, China;

[2]Beijing Entry-exit Inspection and Quarantine Bureau, Beijing 100026, China;

[3]Chinese Society of Inspection and Quarantine, Beijing 100029, China.

*Please address all correspondence to Z. Fan (fanzf@cau.edu.cn)

Supplementary information

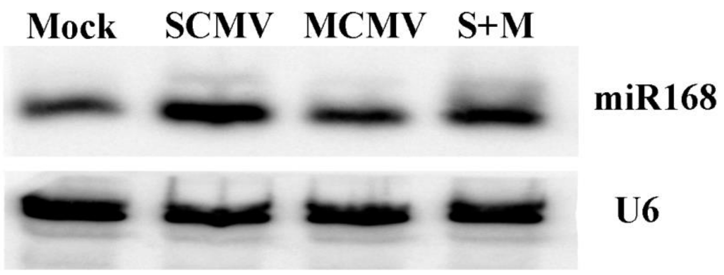

Supplementary Figure S1. The expression level of maize miR168 by Northern blotting analysis. The expression level of maize miR168 was detected by Northern blotting analysis at 9 dpi in Mock, SCMV, MCMV and S+M inoculated maize plants. U6 was used as a loading control.

Supplementary Table S1. The information of vsiRNAs selected for target prediction. The numbers of position represent vsiRNAs starting positions of the (+) sense strand or ending positions of the (-) sense strand of viral genomes.

MCMV				SCMV			
**Sequence**	**Position**	**Strand**	**Counts**	**Sequence**	**Position**	**Strand**	**Counts**
AAAGCCUUGUCGAGACUCUGC	371	+	4610	AAAAUGCGGACAGUGAUUAAC	3377	+	4495
AACCACAGAUUGCAAGAGACC	636	+	3230	AAAUGCGGACAGUGAUUAACAC	3378	+	5974
AACCCUGGGGCAAGUAGAUGCU	4330	+	4027	AACACGAUCGGAAAUGAAUAAG	2611	+	4725
AACCUGUGUACAUCCUUGAACA	2526	+	2735	AACAGAUAAUAGAGGAAGAACC	2304	+	5409
AAGCCUUGUCGAGACUCUGCU	372	+	9464	AACCAUUGACACUAAGUACUG	3486	+	7088
ACAGGGAAAAGACUUUGACAGU	2788	+	2865	AACGGACGAGGAUGAAAUAUU	7174	+	5385
ACGAGGGUUUCUGAACUCAACG	4375	+	4061	AACGGACGAGGAUGAAAUAUUC	7174	+	7323
ACUAAAUCUGAAGGAGCACCUG	3118	+	3086	AAGAGGAUGGCACUGUUAGAAA	965	+	4794
AGAAUGACAUCAAGUCUUGGAC	308	+	2705	AAGAGUUGGAAGGAACAAGCC	4540	+	5182
AGCCAAUGAACUUACGGUUUG	1138	+	2811	AAGUCGAGAUCGAGUCAGCUC	2878	+	7215
AGCCUAAAUCGUAUACUAGUA	4177	+	3401	AAUGCGGACAGUGAUUAACAC	3379	+	5020
AGUUGACUCACGUCUUGCAUCC	47	+	3063	AAUUAUUUCGAAGCUGUGGACC	1517	+	8739
AUACACAAUGGCGGCAAGUAGC	3377	+	5603	ACAGAUAAUAGAGGAAGAACC	2305	+	6362
AUCCGGAAAGCCUUGUCGAGAC	365	+	2574	ACAGGAACAAGAACACCAUCU	8474	+	7847
AUUGGGCAAUUGCAUACUGGCC	2341	+	2653	ACAUACUGGAUUGUCAAGAACC	2405	+	5708
CAAAAGAUCGACAUACACAAUG	3365	+	3166	ACCCUGACAAGAUCUCUGAAU	3960	+	7003
CAACCACAGAUUGCAAGAGACC	635	+	5208	ACCGUGGGACAACUGAUAAAAU	2159	+	10207
CAACGUCCUGAUCUGUCCGGC	2431	+	3239	ACGGAAAUGUCGGCGAGACUC	9240	+	4798
CACGAGGGUUUCUGAACUCAAC	4374	+	14096	ACGGACGAGGAUGAAAUAUUC	7175	+	5704
CACUAAAUCUGAAGGAGCACC	3117	+	3978	ACGGACGAGGAUGAAAUAUUCC	7175	+	5735
CAGGGAAAAGACUUUGACAGU	2789	+	3545	ACGGAGAUUUCUGGAAACACU	6516	+	5484
CAGUUGACUCACGUCUUGCAUC	46	+	3280	ACGGUGAAUGCAGAAGAACUAC	692	+	7465
CAUCGCAAUCAGUUGGAAAAC	3079	+	3138	ACUUCUGUGGUCUCACCUAGA	2581	+	5448
CAUGGACAACACGUACUUACG	4279	+	4881	AGAAAAAGCGUAGAUUUAGGC	2807	+	6054
CCAUGGACAACACGUACUUACG	4278	+	3395	AGAGACAAAGACGUUGACGCU	8621	+	5214
CCGGAACAACCAGUCCUUCUG	4126	+	3003	AGAGUUGGAAGGAACAAGCCC	4541	+	10643
CGAAACAAGUUGACUCGGACU	689	+	2723	AGCAUGUGAUGGAAAUAGAACA	822	+	5371
CGACAUGGUAACUGGAUACACU	4307	+	3341	AGCGGAUACAUUUAGUAAUGAC	3550	+	6276
CGAGACUCUGCUUGACACGGA	381	+	3615	AGGAUGGCACUGUUAGAAAUC	968	+	9006
CUACGGAUUUGAAGUCAUAUC	2500	+	2740	AGGAUGGCACUGUUAGAAAUCC	968	+	6001
CUUGACGAAGACACAGGACACC	3289	+	3486	AUCGGAAAUGAAUAAGGAACU	2617	+	4795
GCCGUGCGAUCUGAAGACAACC	3058	+	4629	AUGGAUACUGUUACAUUAACA	1875	+	4803
GCCUUGUCGAGACUCUGCUUGA	374	+	2550	AUUUCCAGAAGAUCAGAGACU	1626	+	5614
GGCCAAAUCAAAAUCUCGUACU	4002	+	2573	CAAUGUCGAAGAAAAUGCGCC	8679	+	5143
GUAACUGGAUACACUUAACCCU	4314	+	3313	CACGGUGAAUGCAGAAGAACU	691	+	10181
GUGUGUAGAUUCGUCCUGGCC	3214	+	2696	CACUAGUCUCCUGGAAACCCU	9335	+	5238
GUGUGUAGAUUCGUCCUGGCCU	3214	+	3512	CACUGAGAGUUGAUUUGACGCC	5946	+	4709

UAACCACUUCAAUUUCAACUG	3180	+	2829	CACUGUUAGAAAUCCUAUUCC	975	+	4893
UAACCCUGGGGCAAGUAGAUGC	4329	+	2567	CAGCCGUCAACAGUUGUAGAC	7772	+	5065
UAAGAAAGCUGAUCCUGUACC	1918	+	2600	CAGCGGAUACAUUUAGUAAUG	3549	+	4459
UACAAUAGCUCUGAAGAACAGA	3827	+	2750	CAUGGCUCACGGAGUGUAUUC	6852	+	4571
UACACAAUGGCGGCAAGUAGC	3378	+	2911	CGGUCGAACUUACCUGAGCACC	8105	+	5063
UAUCUGAGACCGGUUGAGCACC	1988	+	4006	GGAUGGCACUGUUAGAAAUCC	969	+	4625
UCGAAACAAGUUGACUCGGACU	688	+	3738	UAACGGACGAGGAUGAAAUAUU	7173	+	4756
UCGAGACUCUGCUUGACACGGA	380	+	3373	UACCGUGGGACAACUGAUAAAA	2158	+	4950
UGCGAUCUGAAGACAACCAUCG	3062	+	4685	UAUUUCGAAGCUGUGGACCCA	1520	+	7673
UGCUGACCAGAUUCUCGAGAUU	3566	+	4500	UCUUGGACUCACGUGACAUACA	158	+	7839
UGUGUGUAGAUUCGUCCUGGCC	3213	+	2838	UUAUUUCGAAGCUGUGGACCC	1519	+	6406
UUGACGAAGACACAGGACACC	3290	+	3624	UUAUUUCGAAGCUGUGGACCCA	1519	+	4510
UUGGGCAAUUGCAUACUGGCC	2342	+	3992	UUUCCAGAAGAUCAGAGACUC	1627	+	4893
AAACACUUUGGAUUGGCAGGAC	4152	-	1960	AACGAGUCUCUGAUCUUCUGGA	1629	-	7228
AAGACAUUCGGGAAUUUCUGCC	2980	-	1946	AAGCCUGUGGAUUUUCCUGACC	3741	-	5561
ACACGGAGUACGAGAUUUUGA	4009	-	2082	AAGUGAAUCAUGCGUAGAAGUU	1134	-	6162
ACACGGAGUACGAGAUUUUGAU	4008	-	2189	AAUAGGAUUUCUAACAGUGCC	973	-	5859
ACAGAUCAGGACGUUGUCAUC	2426	-	4319	AAUGAGAUUUGGGUUGUAAUGC	6718	-	4232
ACCAGCCUGUAUACACCUGAAC	3884	-	2114	ACAGAGAGUGCAUGUUGCGAC	9297	-	5109
ACGAUUUAGGCUCCCAAACACU	4167	-	1687	ACCGUGCUGUAGCAUUUCGCA	676	-	5848
ACUAGUAUACGAUUUAGGCUCC	4175	-	5281	ACGAAGCAUUUCUUACUGGAUG	4362	-	4356
AGAACGUGUUUAGUCAGACAGU	975	-	1937	ACGAGUCUCUGAUCUUCUGGA	1629	-	4187
AGACAUUCGGGAAUUUCUGCC	2980	-	2622	AGACUUUGUAGUUCUUCUGCAU	699	-	4172
AGAUGAACAGAAUCGAGGAGAU	3269	-	2175	AGAUUGCUGUAUAACUUGGCC	1066	-	4627
AGCAGAAUUUCGGACAUGGAGC	3526	-	6582	AGAUUGCUGUAUAACUUGGCCC	1065	-	5566
AGCCUGUAUACACCUGAACACA	3881	-	2670	AGCCUGUGGAUUUUCCUGACC	3741	-	5759
AGGACCAAACUCACGGAUGAGA	1054	-	2590	AGCCUGUGGAUUUUCCUGACCC	3740	-	5242
AUACCGUGACGUUUGUUGAAUC	721	-	5712	AGUAAAUACGAGGUAGAACCU	9421	-	4544
AUAGCCACAAUGAAUCGUCCUG	3952	-	1892	AGUAGAAUUGGUUGUUGAAAUC	7454	-	4951
AUCGGAUUUUAGCUCCCACCU	833	-	1748	AGUAUUAACUUUGAUGAGCGCC	1270	-	6283
AUGAACAGAAUCGAGGAGAUC	3268	-	2106	AGUGAAUCAUGCGUAGAAGUU	1134	-	8247
AUGCAGCAAACUGACCAGCCU	3898	-	1806	AGUUCCAGAUCAUCAAUAUUG	1027	-	4989
CAACGAUCUGUGAACAUAGCU	456	-	2153	AUAGUAAAUACGAGGUAGAAC	9423	-	4303
CACAAUGAAUCGUCCUGGGGA	3948	-	1855	AUAGUAAAUACGAGGUAGAACC	9422	-	4704
CACAUCUGUGAUUGUGCUGCC	3656	-	2058	AUCCCUGAUCAAUUUCGUGACG	460	-	11843
CACGGAGUACGAGAUUUUGAU	4008	-	1883	AUGGCACAUUCUGUUCGAUUG	3399	-	4888
CACGGAGUACGAGAUUUUGAUU	4007	-	2142	AUUGCUGUAUAACUUGGCCCC	1064	-	10062
CAGAAUUUCGGACAUGGAGCA	3525	-	4383	CACCAAGAACUUCCUCUGAUC	5619	-	5488
CCAUUGUUGAUUAAUCUGGCU	2404	-	2470	CACCAAGAACUUCCUCUGAUCC	5618	-	5354
CCGCGAUUAUGAAUCUGCCACC	2244	-	2196	CACCGUGCUGUAGCAUUUCGCA	676	-	4465
CGAACUGCUAGCCGAACAUACC	738	-	1907	CAGAUUGCUGUAUAACUUGGC	1067	-	6505
CGAGGACACAUGAAAUAUAUGC	782	-	2020	CAGUUCCAGAUCAUCAAUAUUG	1027	-	4130
CGCGAUUAUGAAUCUGCCACC	2244	-	5575	CCAUAGCAACUUGUAGGGCUG	5017	-	4241
CGGACAGAUCAGGACGUUGUC	2429	-	2092	CGCUGGAUCGACAUGAUUAAG	2555	-	4889
CUACUAGUAUACGAUUUAGGCU	4177	-	2007	CUUCCAAGUCUCUGAAUACGCU	4521	-	5844
CUAGUAUACGAUUUAGGCUCC	4175	-	2143	GUAGAAUUGGUUGUUGAAAUC	7454	-	7176

CUGGAAUCUCGAGAAUCUGGUC	3570	-	2142	GUAUUAACUUUGAUGAGCGCC	1270	-	7166
GAGGACACAUGAAAUAUAUGCC	781	-	2033	UACAACUGUUGACGGCUGCCC	7769	-	4847
GCAGAAUUUCGGACAUGGAGC	3526	-	1936	UACACCGAUCUUUGACAAAGU	1152	-	5141
GUCCUGUGUCUUCGUCAAGAUG	3286	-	3725	UAGGUUUCCAAAGUGAUUUUG	7946	-	4883
GUUCCGCGAUUAUGAAUCUGCC	2247	-	1761	UAUCAACAUCCAGUUCGGCUGU	4457	-	5528
UACCGUGACGUUUGUUGAAUC	721	-	2326	UAUUCCAACUGUCCAAGGACC	7490	-	7122
UACUAGUAUACGAUUUAGGCU	4177	-	2370	UCAAACUUUACUAGAUCGGCC	4759	-	6424
UAGACAUACUCUUUCAAUGCCU	810	-	3435	UCCAUAGCAACUUGUAGGGCU	5018	-	4747
UAGUAUACGAUUUAGGCUCCC	4174	-	3384	UCGCUGGAUCGACAUGAUUAAG	2555	-	7970
UCGCACCGUUCGUAAGUACGUG	4288	-	3609	UGAUAUUGGAGUGUAGUCACC	6134	-	4406
UGAUCGGAUUUUAGCUCCCACC	834	-	2154	UUACACCGAUCUUUGACAAAG	1153	-	4175
UGCAACGAUCUGUGAACAUAGC	457	-	1991	UUACACCGAUCUUUGACAAAGU	1152	-	5070
UGCAAGACGUGAGUCAACUGUA	44	-	1708	UUACUAGAUCGGCCAUUACAAA	4751	-	4104
UGCAAGCAGAAUUUCGGACAUG	3530	-	1920	UUCCAUAGCAACUUGUAGGGCU	5018	-	6387
UGUCGCACCGUUCGUAAGUACG	4290	-	2112	UUGCGGAUUCCUCAUUGACAUU	1916	-	3980
UGUCUGAUCGAUCGAGUACACC	3334	-	2428	UUGCUGUAUAACUUGGCCCCA	1063	-	4865
UUCGAGUAGACAUCCACAGAC	672	-	1609	UUUUGUUGAGAACCUUGUAAAC	3359	-	6244

Supplementary Table S2. The predicted mRNA targets of the selected vsiRNAs in maize.

vsiRNA sequence	Target ID	Position	Max Score	Max Energy
AAGUCGAGAUCGAGUCAGCUC	GRMZM2G096363_T01	1117	191	-38.91
AACAGAUAAUAGAGGAAGAACC	AC188752.3_FGT008	271	188	-24.86
UAUCAACAUCCAGUUCGGCUGU	AC209208.3_FGT004	1312	188	-27.09
AGUAGAAUUGGUUGUUGAAAUC	GRMZM2G304274_T01	2791	188	-25.12
AGUAGAAUUGGUUGUUGAAAUC	GRMZM2G123277_T01	2845	188	-25.12
ACUUCUGUGGUCUCACCUAGA	GRMZM2G009443_T01	840	187	-33.08
AAUUAUUUCGAAGCUGUGGACC	GRMZM2G109315_T01	1131	184	-21.29
AAAUGCGGACAGUGAUUAACAC	GRMZM2G113888_T01	1981	184	-26.23
CGGUCGAACUUACCUGAGCACC	GRMZM2G064875_T03	197	184	-33.9
UUUUGUUGAGAACCUUGUAAAC	GRMZM2G128809_T01	458	184	-23.16
AAGCCUGUGGAUUUUCCUGACC	GRMZM2G160279_T01	973	184	-26.18
UAUCAACAUCCAGUUCGGCUGU	GRMZM2G006178_T01	724	184	-26.77
CACCAAGAACUUCCUCUGAUCC	GRMZM2G025342_T01	1057	184	-26.16
AAUGAGAUUUGGGUUGUAAUGC	GRMZM2G026020_T01	509	184	-30.72
AGACUUUGUAGUUCUUCUGCAU	GRMZM2G704239_T01	266	184	-24.88
CAGUUCCAGAUCAUCAAUAUUG	GRMZM2G109843_T01	2197	184	-28.58
CAGUUCCAGAUCAUCAAUAUUG	GRMZM2G109843_T02	2352	184	-28.58
ACAGAUAAUAGAGGAAGAACC	GRMZM2G118957_T04	816	183	-25.56
ACAGAUAAUAGAGGAAGAACC	AC188752.3_FGT008	271	183	-25.31
AACAGAUAAUAGAGGAAGAACC	AC206780.3_FGT004	1066	183	-23.84
AACAGAUAAUAGAGGAAGAACC	GRMZM2G325633_T01	1387	183	-23.84
CACUAGUCUCCUGGAAACCCU	GRMZM2G033478_T01	2534	183	-34
CACUAGUCUCCUGGAAACCCU	GRMZM2G033478_T02	2534	183	-34

GUAGAAUUGGUUGUUGAAAUC	GRMZM2G304274_T01	2791	183	-25.92
GUAGAAUUGGUUGUUGAAAUC	GRMZM2G123277_T01	2845	183	-25.92
GUAGAAUUGGUUGUUGAAAUC	GRMZM2G118462_T01	2975	183	-23.4
UUUUGUUGAGAACCUUGUAAAC	GRMZM2G098039_T01	341	183	-21.52
CUUCCAAGUCUCUGAAUACGCU	GRMZM2G100709_T01	608	183	-26.12
ACAGAGAGUGCAUGUUGCGAC	GRMZM2G057176_T01	24	183	-30.99
CCAUAGCAACUUGUAGGGCUG	GRMZM2G082257_T01	890	183	-24.79
CCAUAGCAACUUGUAGGGCUG	GRMZM2G082257_T03	864	183	-24.79
AGACUUUGUAGUUCUUCUGCAU	GRMZM2G035153_T01	1025	183	-25.8
AGACUUUGUAGUUCUUCUGCAU	GRMZM5G832989_T01	1515	183	-26.88
AGACUUUGUAGUUCUUCUGCAU	GRMZM5G832989_T02	1509	183	-26.88
AGACUUUGUAGUUCUUCUGCAU	GRMZM5G892361_T01	915	183	-25.8
AGACUUUGUAGUUCUUCUGCAU	GRMZM5G892361_T02	1011	183	-25.8
AUUUCCAGAAGAUCAGAGACU	GRMZM2G383154_T01	459	182	-25.11
AUUUCCAGAAGAUCAGAGACU	GRMZM2G383154_T02	675	182	-25.11
AUUUCCAGAAGAUCAGAGACU	GRMZM2G383154_T04	459	182	-25.11
ACGGAGAUUUCUGGAAACACU	GRMZM2G420108_T02	38	182	-28.24
AGCAUGUGAUGGAAAUAGAACA	GRMZM2G063961_T01	608	182	-25.46
CAAUGUCGAAGAAAAUGCGCC	GRMZM2G086093_T01	735	182	-23.74
CAAUGUCGAAGAAAAUGCGCC	GRMZM2G086093_T02	735	182	-23.74
CAAUGUCGAAGAAAAUGCGCC	GRMZM2G086093_T03	508	182	-23.74
CACUGAGAGUUGAUUUGACGCC	GRMZM2G408379_T02	2582	182	-25.74
CACCAAGAACUUCCUCUGAUC	GRMZM2G025342_T01	1058	182	-25.58
CACCAAGAACUUCCUCUGAUCC	GRMZM2G017528_T01	1230	182	-39.39
UUGCGGAUUCCUCAUUGACAUU	GRMZM5G824201_T02	582	182	-33.16
ACAGGAACAAGAACACCAUCU	GRMZM2G059042_T01	1604	181	-28.46
ACCCUGACAAGAUCUCUGAAU	GRMZM2G322490_T01	659	181	-32.31
ACGGACGAGGAUGAAAUAUUCC	GRMZM2G131254_T01	89	181	-28.9
ACGGACGAGGAUGAAAUAUUCC	GRMZM2G131254_T02	89	181	-28.9
ACGGACGAGGAUGAAAUAUUCC	GRMZM2G131254_T03	89	181	-28.9
ACGGACGAGGAUGAAAUAUUCC	GRMZM2G131254_T04	89	181	-28.9
ACGGACGAGGAUGAAAUAUUCC	GRMZM2G131254_T05	89	181	-28.9
ACGGACGAGGAUGAAAUAUUC	GRMZM2G131254_T01	90	181	-28.9
ACGGACGAGGAUGAAAUAUUC	GRMZM2G131254_T02	90	181	-28.9
ACGGACGAGGAUGAAAUAUUC	GRMZM2G131254_T03	90	181	-28.9
ACGGACGAGGAUGAAAUAUUC	GRMZM2G131254_T04	90	181	-28.9
ACGGACGAGGAUGAAAUAUUC	GRMZM2G131254_T05	90	181	-28.9
AUUUCCAGAAGAUCAGAGACU	GRMZM2G118453_T01	93	181	-28.55
CACUAGUCUCCUGGAAACCCU	GRMZM2G108716_T01	901	181	-33.28
CACUAGUCUCCUGGAAACCCU	GRMZM2G108716_T02	1042	181	-33.28
CACCAAGAACUUCCUCUGAUC	GRMZM2G017528_T01	1231	181	-35.87
AAUUAUUUCGAAGCUGUGGACC	GRMZM5G848602_T01	844	180	-24.08
UCUUGGACUCACGUGACAUACA	GRMZM2G174147_T01	284	180	-27.11
ACGGUGAAUGCAGAAGAACUAC	GRMZM2G020151_T02	1635	180	-24.63
AACAGAUAAUAGAGGAAGAACC	GRMZM2G106042_T01	1114	180	-20.63
AACAGAUAAUAGAGGAAGAACC	GRMZM2G106042_T02	1293	180	-20.63

AACAGAUAAUAGAGGAAGAACC	GRMZM2G106042_T03	1273	180	-20.63
AACAGAUAAUAGAGGAAGAACC	GRMZM2G106042_T04	1278	180	-20.63
AACAGAUAAUAGAGGAAGAACC	GRMZM2G106042_T05	1258	180	-20.63
AAGAGUUGGAAGGAACAAGCC	GRMZM2G128319_T01	1817	180	-27.58
AAGAGGAUGGCACUGUUAGAAA	GRMZM2G155242_T01	1528	180	-24.65
AAGAGGAUGGCACUGUUAGAAA	GRMZM2G155242_T02	539	180	-24.65
AUCCCUGAUCAAUUUCGUGACG	GRMZM2G051327_T01	945	180	-25.21
AUCCCUGAUCAAUUUCGUGACG	GRMZM2G407825_T01	1575	180	-29.58
AACGAGUCUCUGAUCUUCUGGA	GRMZM2G112229_T01	1228	180	-36.67
CUUCCAAGUCUCUGAAUACGCU	GRMZM2G140082_T02	889	180	-22.29
AAGCCUGUGGAUUUUCCUGACC	AC212112.4_FGT002	893	180	-26.95
AAGCCUGUGGAUUUUCCUGACC	GRMZM2G180815_T01	709	180	-25.5
AAGCCUGUGGAUUUUCCUGACC	GRMZM2G420694_T01	481	180	-25.5
AAGCCUGUGGAUUUUCCUGACC	GRMZM2G015959_T01	976	180	-25.5
CACCAAGAACUUCCUCUGAUCC	GRMZM5G832772_T01	3576	180	-27.86
CACCAAGAACUUCCUCUGAUCC	GRMZM5G832772_T02	3576	180	-27.86
AGCCUGUGGAUUUUCCUGACCC	GRMZM2G101000_T02	784	180	-31.08
AGUUCCAGAUCAUCAAUAUUG	GRMZM2G157598_T01	2139	180	-27.89
AGUAGAAUUGGUUGUUGAAAUC	GRMZM2G072886_T01	997	180	-23.55
AGUAGAAUUGGUUGUUGAAAUC	GRMZM2G072886_T02	996	180	-23.55
AGUAGAAUUGGUUGUUGAAAUC	GRMZM2G020852_T01	877	180	-23.55
AGUAGAAUUGGUUGUUGAAAUC	GRMZM2G146551_T01	691	180	-23.55
AGUAGAAUUGGUUGUUGAAAUC	GRMZM2G146551_T02	877	180	-23.55
AGUAGAAUUGGUUGUUGAAAUC	GRMZM2G181202_T01	877	180	-23.55
AGUAGAAUUGGUUGUUGAAAUC	GRMZM2G181202_T02	691	180	-23.55
CACCGUGCUGUAGCAUUUCGCA	GRMZM2G128564_T01	1410	180	-36.56
CACCGUGCUGUAGCAUUUCGCA	GRMZM2G128564_T02	878	180	-36.56
AGACUUUGUAGUUCUUCUGCAU	GRMZM2G440695_T01	2821	180	-22.34
CAGUUCCAGAUCAUCAAUAUUG	GRMZM2G700386_T01	294	180	-26.64
CAGUUCCAGAUCAUCAAUAUUG	GRMZM2G137820_T02	1449	180	-26.64
CAGUUCCAGAUCAUCAAUAUUG	GRMZM2G108892_T01	956	180	-30.24

Supplementary Table S3. The probes for Northern blotting of small RNAs.

vsiRNA sequence	Probe name	Probe sequence
AAGCCUUGUCGAGACUCUGCU	M372(+)	AGCAGAGTCTCGACAAGGCTT
AAG AUCGACAUAC ACAAUGGC	M3368(+)	GCCATTGTGTATGTCGATCTT
AACCCUGGGGC AAGUAGAUGC	M4330(+)	GCATCTACTTGCCCCAGGGTT
ACGAGG GUUUCUGAAC UCAAC	M4375(+)	GTTGAGTTCAGAAACCCTCGT
UAGACAUACUCUUUCAAUGCC	M831(-)	GGCATTGAAAGAGTATGTCTA
GCAGAAUUUC GGACAUGGAGC	M3546(-)	GCTCCATGTCCGAAATTCTGC
CACGGAGUAC GAGAUUUUGAU	M4028(-)	ATCAAAATCTCGTACTCCGTG
ACUAGUAUAC GAUUUAGGCUC	M4196(-)	GAGCCTAAATCGTATACTAGT
CGGUGAAUGCAGAAGAACUAC	S693(+)	AGTTCTTCTGCATTCACCG

AAGAGUUGGAAGGAACAAGCC	S4541(+)	GGCTTGTTCCTTCCAACTCT
GUUGAGAGAGAAGAAUCAGAGA	S109(-)	TCTCTGATTCTTCTCTCTCAAC
UUCCAUAGCAACUUGUAGGGC	S5017(-)	GCCCTACAAGTTGCTATGG
	Zma-miR168-probe	GTCCCGATCTGCACCAAGCGA
	U6-probe	CGATTTGTGCGTGTCATCCTTG

Supplementary Table S4. The primers for qRT-PCR of maize *DCLs* and several *AGOs* mRNAs.

Gene name	Gene ID	Primer name	Primer sequence
DCL1[▲]	GRMZM2G040762_T01	DCL1-F	TGGTTGATGGCATACAGATTGG
		DCL1-R	CAAGCAGATGTCGTTCAGAGTC
DCL2[▲]	GRMZM2G301405_T01	DCL2-F	CCAGTTCCGTGCTCCGTGAT
		DCL2-R	CAGTAAAAGTAGGGTAGCGTGCC
DCL3a[▲]	AC194839.3_FGT008	DCL3a-F	GGCGTGCGGATAAGAAGAG
		DCL3a-R	GACTGATTTGGCGGTGACAT
DCL3b[▲]	GRMZM2G413853_T01	DCL3b-F	CTTGCGTTGCCACCTTATCG
		DCL3b-R	TGTCCTTGTGCTATCAGTTGTTCA
DCL4[▲]	GRMZM2G160473_T01	DCL4-F	TCGGAACTGATAGGCTATGATGAA
		DCL4-R	CCTCTAGGTAGGCAACCATTCT
AGO1a[★]	GRMZM2G441583_T01	AGO1a-F	ACACCGTTATGCACCGGTAA
		AGO1a-R	AATCCACGACAAGCCCATCTT
AGO1b	GRMZM2G039455_T01	AGO1b-F	TTACCAAAGCATAAGGCCGACAC
		AGO1b-R	CTTCACGCGATCAGAATCAGACA
AGO1c[★]	AC209206.3_FTG011	AGO1c-F	CTACCATGTCCTGTGGGACG
		AGO1c-R	GCTGCCAGATGAGCGTAGT
AGO2a[▲]	GRMZM2G007791_T01	AGO2a-F	CTTACCTGCCAGCCAGATGACCTT
		AGO2a-R	TGTCCTTGCTCTTGCTCAAATCCA
AGO18a	GRMZM2G105250_T01	AGO18a-F	CCAGCATACTACGCCCACAA
		AGO18a-R	ATGAGCCTTCTCAGTTCACCCT

"▲" means the primers referred to Xia *et al.*

"★" means the primers referred to Zhai *et al.*
